# Editorial: Genetics and Genomics of Polyploid Plants

**DOI:** 10.3390/genes15111377

**Published:** 2024-10-25

**Authors:** Nunzio D’Agostino, Carlo Fasano

**Affiliations:** 1 Department of Agricultural Sciences, University of Naples Federico II, 80055 Portici, Italy; 2 Italian National Agency for New Technologies, Energy and Sustainable Economic Development, Trisaia Research Centre, 75026 Rotondella, Italy

Polyploidy, the condition of having more than two complete sets of chromosomes, is a widespread and influential phenomenon in the plant kingdom. It has been pivotal in shaping plant evolution, driving biodiversity, and facilitating domestication and improvement in crops. Whole-genome polyploidization events are central to this process, as polyploid plants often display unique phenotypes, increased vigor, and enhanced adaptability to environmental conditions. These advantages confer a fitness edge to polyploids over their diploid counterparts, influencing a wide array of traits, including morphology, physiology, and stress resilience [1,2]. Additionally, the genetic complexity of polyploid genomes supports the production of hybrid proteins and fosters protein diversity, creating a dynamic landscape for evolutionary innovation [3].

Polyploidization occurs through two principal mechanisms: autopolyploidy, which results from genome duplication within a single species, and allopolyploidy, arising from hybridization between different species followed by genome doubling. These genome duplication events act as significant genomic shocks, initiating widespread genome rearrangements, alterations in gene regulation, and shifts in the epigenetic landscape [4,5]. Furthermore, polyploidization stimulates transposable element activity, driving additional genomic changes that contribute to the evolutionary trajectory of polyploids [6].

Recent research continues to reveal the complexity of polyploid genomes and the diverse ways polyploidy affects plant evolution and adaptation. Advances in genomics and multi-omics approaches have deepened our understanding of how polyploidization reshapes plant biology at the molecular, physiological, and ecological levels, underscoring its role as a powerful evolutionary force in the plant kingdom [7].

Genome-wide association studies (GWAS) and genomic prediction are increasingly vital in the study of polyploid plants, providing insights into the genetic basis of complex traits and aiding in enhancing breeding strategies. GWAS allow researchers to identify single nucleotide polymorphisms (SNPs) associated with desirable traits, facilitating the selection of superior individuals in breeding programs [8].

Genomic prediction, on the other hand, utilizes high-density SNP data to estimate the genetic potential of untested individuals, offering a powerful tool to accelerate breeding programs and enhance selection efficiency [9]. By integrating genetic information with advanced statistical models, genomic prediction has demonstrated significant potential in improving the accuracy of trait predictions, especially for complex traits. Recent advancements in these models have further enhanced their precision, providing breeders with more reliable insights into selecting individuals with superior traits, ultimately speeding up crop improvement efforts.

The paper by Angelin-Bonnet et al. [10] introduces the Hidecan R package, a tool designed to enhance the visualization of results from GWAS alongside differential expression analyses.

The authors emphasize that the integration of these two types of genomic data can provide deeper insights into the genetic mechanisms underlying phenotypic variation.

Hidecan is integrated into the VIEWpoly tool (https://github.com/mmollina/viewpoly, accessed on 16 October 2024), which is a Shiny app and R package for visualizing and exploring results from polyploid computational tools using an interactive graphical user interface. Hidecan generates comprehensive visual representations which allow users to simultaneously view GWAS peaks and differentially expressed genes. This integrated approach facilitates the identification of potential gene targets related to specific traits and enhances the interpretation of complex datasets. The authors showcase the package capabilities through practical examples, demonstrating its effectiveness in making genomic data more accessible and interpretable.

Additionally, the paper discusses the potential applications of Hidecan beyond GWAS and differential expression studies, suggesting that it could be adapted for other genomic assays. The authors provide a user-friendly interface and thorough supporting information to assist researchers in implementing the package effectively in their analyses.

The paper by García-Barrios et al. [11] focuses on enhancing genomic prediction models for wheat by utilizing data gathered from multi-environment trials. The researchers demonstrated that integrating environmental data into genomic models significantly improves the accuracy of predicting phenotypic traits such as yield and disease resistance in wheat varieties. A key finding is the effectiveness of machine learning techniques in analyzing complex datasets from these trials. By leveraging such advanced methodologies, the authors successfully identified important genetic markers that correlate with desirable traits, providing breeders with valuable insights for selection.

This study underscores the importance of environmental factors in the growth and performance of wheat, suggesting that a comprehensive approach to data analysis can optimize breeding strategies. This can ultimately lead to the development of wheat varieties that are better adapted to diverse environmental conditions, enhancing agricultural sustainability.

The study by Cuevas et al. [12] examines the role of epistasis within and between the three sub-genomes of synthetic hexaploid wheat (*Triticum aestivum*). The research aimed to enhance the prediction of disease resistance traits by analyzing the contributions of different genetic interactions, particularly focusing on the D sub-genome.

The authors employed four statistical models to assess the influence of both additive genetic effects and epistatic interactions, which include both intra- and inter-sub-genomic effects. The findings revealed that epistasis accounted for a significant portion of the genetic variation related to three wheat diseases: tan spot, septoria nodorum blotch, and spot blotch. Specifically, the study highlighted that the D sub-genome plays a crucial role in these disease traits, contributing approximately 50% of the variance for septoria nodorum blotch and spot blotch, while its influence was slightly lower for tan spot at 29%. Furthermore, the predictive accuracy of the models improved notably when epistatic interactions were considered. The best model, which included both additive effects and epistasis (ABDI model), showed an increase in predictive ability compared to models considering only additive effects. This enhancement in prediction accuracy underscores the importance of incorporating complex genetic interactions in genomic selection for disease resistance in wheat. Overall, the study suggests that future breeding strategies could benefit from a focused selection of parental crosses based on the distinct contributions of each sub-genome, especially the D sub-genome, to improve disease resistance traits in wheat.

Transcriptomics studies play a pivotal role in understanding the complexity and adaptability of polyploid plants. By analyzing gene expression profiles in polyploids, researchers can identify how these organisms regulate genes in response to environmental stresses, developmental cues, and metabolic demands. Additionally, transcriptomics can facilitate the development of targeted breeding strategies, ultimately improving crop productivity and sustainability in the face of climate change [13,14,15].

The study by Du et al. [16] investigates how lignin biosynthesis influences the formation of storage roots in hexaploid sweet potatoes. The researchers performed transcriptomic analyses on two sweet potato cultivars, Jishu25 and Jishu29, at different stages of root development. Key findings include the identification of genes involved in lignin biosynthesis that appear to play a significant role in the early stages of storage root formation. This study also highlighted the differential expression of genes between the two cultivars, which helped pinpoint the key regulatory factors that affect the yield of sweet potato storage roots. By understanding these genetic mechanisms, the research contributes valuable insights into improving sweet potato breeding for higher yields and better root formation.

The paper by Aufiero et al. [17] explores the complexities of gene expression in allopolyploid plants. The authors emphasize that transcriptomic analysis is crucial for understanding how duplicated genes in allopolyploids evolve and adapt, leading to novel traits. However, the redundancy created by multiple gene copies complicates the analysis. They discuss the need for sub-genome phasing and the inference of homoeologs—similar genes from different genomes—to achieve accurate gene expression profiling. This paper advocates for bulk RNA sequencing as a primary method for studying these processes. It highlights the importance of proper read mapping and normalization to effectively analyze gene expression, enabling the identification of subtle expression differences that are key to understanding the regulatory dynamics in allopolyploids.

Allele mining is crucial for harnessing genetic diversity in crops, as it enables the identification of beneficial alleles associated with important agronomic traits. This process facilitates the discovery of novel alleles that can enhance stress tolerance, yield, and quality. By leveraging the increased genetic variability found in polyploids, researchers can develop more resilient and productive varieties tailored to changing environmental conditions. Furthermore, integrating allele mining with genomic tools enhances the efficiency of marker-assisted selection, allowing breeders to make informed decisions based on the genetic potential of different alleles.

The review by Afshari-Behbahanizadeh et al. [18] investigates the critical role of vernalization in wheat reproductive growth and how variations in Vrn genes impact this process. Vernalization is essential for the transition from vegetative to reproductive growth in wheat, significantly affecting flowering time, grain filling, and overall yield, especially in the context of climate change that alters winter and spring temperatures.

The authors highlight the structural complexity and functional diversity of different alleles associated with the Vrn genes (Vrn-1, Vrn-2, Vrn-3, and Vrn-4) across various wheat species, including diploid, tetraploid, and hexaploid forms. They detail the molecular mechanisms and allelic variations that influence flowering time, emphasizing the importance of Vrn-1 alleles. This review also addresses inconsistencies in the nomenclature for these alleles, which can hinder collaborative research efforts. Furthermore, this study underscores the potential for utilizing genome and transcriptome sequencing advancements to better understand Vrn gene functions and evolutionary patterns. This knowledge could lead to innovative strategies for modifying flowering time and enhancing wheat productivity, especially in changing environmental conditions.

In summary, the studies highlighted in this editorial illuminate the intricate relationships between genetic mechanisms, environmental factors, and plant phenotypes across various species, particularly in wheat, while collectively enhancing our understanding of the genetic foundations of key agricultural traits. This research paves the way for improved breeding strategies and strengthen crop resilience in the face of environmental changes, promising to inform future research and practical applications in plant genetics and agriculture.
